# Altered GABA_B_ receptor sensitivity in heavy drinkers indexed using a TMS-based biomarker: a randomized controlled experimental medicine trial

**DOI:** 10.1038/s41398-026-04530-4

**Published:** 2026-10-23

**Authors:** Chiara Montemitro, Daphne Donahue, Andrea Costumati, Federico Maruottolo, Sara Terezza, Caroline Sjoberg, Irene Perini, Markus Roman, Fredrik C. Kugelberg, Markus Heilig

**Affiliations:** 1 Center for Social and Affective Neuroscience, Linköping University, Linköping, Sweden; 2 Department of Psychiatry, University of Parma, Parma, Italy; 3 Department of Forensic Genetics and Forensic Toxicology, National Board of Forensic Medicine, Linköping, Sweden; 4 Division of Clinical Chemistry and Pharmacology, Department of Biomedical and Clinical Sciences, Linköping University, Linköping, Sweden; 5 Present Address: Department of Neuroscience and Rehabilitation, University of Ferrara, Ferrara, Italy

**Keywords:** Diagnostic markers, Physiology, Addiction

## Abstract

Metabotropic γ-aminobutyric acid type B receptors (GABA_B_Rs) are implicated in alcohol use disorder (AUD) and represent a promising therapeutic target. However, robust biomarkers of GABA_B_Rs target engagement and sensitivity in humans are lacking. We examined transcranial magnetic stimulation (TMS)–derived cortical inhibition and growth hormone (GH) release as measures of GABA_B_R function, and whether heavy alcohol use is associated with altered responses to pharmacological GABA_B_R activation. In a randomized, double-blind, placebo-controlled crossover study, 31 healthy volunteers (16 heavy drinkers, 15 light drinkers) received baclofen 30 mg, baclofen 60 mg, or placebo across three sessions. Long-interval intracortical inhibition (LICI), short-interval intracortical inhibition, and intracortical facilitation were assessed with paired-pulse TMS at baseline and ~60 min post-dose. Baclofen blood concentrations and plasma GH levels were measured. Baclofen produced dose-dependent increases in GH release, confirming neuroendocrine GABA_B_R engagement. The LICI response to baclofen differed between groups: In light drinkers, baclofen at 30 mg potentiated LICI, and a similar potentiation was seen at 60 mg, indicating that a plateau may have been reached. In contrast, in heavy drinkers, a potentiation of LICI similar to that in light drinkers was seen at 30 mg baclofen, but was absent at the 60 mg dose despite comparable pharmacokinetics, suggesting altered GABA_B_R function. Effects were selective for GABA_B_R–mediated inhibition, with no consistent modulation of short-interval intracortical inhibition or corticospinal excitability. Acute baclofen administration reveals altered GABA_B_R sensitivity in heavy drinkers, with preserved responses at lower doses but reduced response under stronger agonist stimulation. LICI and GH release provide complementary biomarkers of central and peripheral GABA_B_Rs engagement and may support translational development of mechanism-based treatments for AUD.

## Introduction

Alcohol use disorder (AUD) accounts for ~5% of the worldwide disease burden [1]. Available AUD medications have limited efficacy and patient acceptance [2, 3], and fewer than one-quarter of people with AUD receive pharmacological treatment, often after long delays [4]. This highlights the need for mechanism-based treatments, supported by objective biomarkers of target engagement and individual sensitivity.

Dysregulation of γ-aminobutyric acid (GABA) neurotransmission is a central feature of AUD. Metabotropic GABA_B_ receptors (GABA_B_Rs) are of particular interest, due to their widespread expression at pre- and postsynaptic sites, and their role in regulating neuronal excitability, neurotransmitter release, and reward-related circuitry [5–7]. Chronic alcohol exposure induces neuroadaptations in GABA_B_R signaling within cortico-limbic networks, contributing to impaired inhibitory control and compulsive alcohol use [7, 8].

Preclinical research has demonstrated a causal role for altered GABAergic transmission in alcohol addiction-related behaviors [9–12]. For instance, in outbred rats, a vulnerable minority preferentially chooses alcohol over a natural reward, and persists in alcohol taking despite adverse consequences. This is associated with reduced GABA transporter expression and altered GABA tone in the central amygdala, changes also observed in post-mortem brain tissue from people with AUD [10, 13]. Activation of GABA_B_Rs rescues both excessive alcohol choice and compulsive self-administration in these animals, mechanistically validating GABA_B_Rs as a treatment target [13, 14].

Early clinical studies with baclofen, the only GABA_B_R agonist approved for human use, suggested its efficacy in AUD [15]. Meta-analysis of subsequent trials also provided support for this effect, but results have been heterogeneous, and clinical use is limited by tolerance and dose-related adverse effects [16]. Positive allosteric GABA_B_R modulators (PAMs) may overcome these limitations, offering a path forward for therapeutics targeting GABA_B_Rs. Similar to baclofen, GABA_B_R PAMs suppress alcohol intake in preclinical models, underscoring the translational relevance of this mechanism, and the need for biomarkers of GABA_B_R target engagement in humans [9, 12]

Transcranial magnetic stimulation (TMS) combined with electromyography provides a non-invasive approach to probe inhibitory and excitatory cortical circuits in vivo. Paired-pulse TMS paradigms allow dissociation of distinct neurochemical processes [17]. Long-interval intracortical inhibition (LICI) reflects postsynaptic GABA_B_R–mediated cortical inhibition and is potentiated by baclofen in healthy individuals [18, 19]. In contrast, short-interval intracortical inhibition (SICI) and intracortical facilitation (ICF) index GABA_A_- and glutamatergic mechanisms, respectively, although they may also be indirectly modulated by GABA_B_R activity [20]. Our recent systematic review of TMS studies in AUD indicates that GABA_B_R-mediated measures of cortical inhibition — particularly LICI — are altered following both acute and chronic alcohol exposure, and differ between people with AUD and healthy controls, supporting their potential as biomarkers of GABA_B_Rs dysfunction [21].

GABA_B_Rs also regulate growth hormone (GH) secretion by the hypothalamic–pituitary axis. Baclofen-induced GH release is an established pharmacodynamic biomarker of GABA_B_R activation in healthy individuals, whereas this response is blunted or absent in AUD, and not fully restored by abstinence [22–24]. Although the precise mechanisms remain incompletely understood, this response has been proposed to predominantly reflect activation of presynaptic GABAergic pathways regulating hypothalamic hormone secretion. To our knowledge, despite convergent findings suggesting the imporance of this mechanism, no study has examined GABA_B_R function in humans as a function of alcohol use severity, and compared TMS-derived measures of cortical inhibition with GH responses.

Here, we carried out a randomized, double-blind, placebo-controlled crossover trial designed to address this gap. We investigated the acute effects of different baclofen doses (30 and 60 mg) on intracortical inhibition and facilitation, alongside plasma baclofen concentrations and GH release, in heavy (HD) and light (LD) drinkers. This approach aimed to establish translational pharmacodynamic biomarkers of GABA_B_R target engagement, and to determine whether biomarker responsiveness varies across different levels of alcohol consumption. Non-treatment-seeking heavy drinkers represent an established human experimental medicine model for investigating early alcohol-related neurobiological adaptations while minimizing the potential confounding effects of withdrawal, psychiatric comorbidities and concomitant medications [25, 26].

## Methods

### Design

**Fig. 1:**
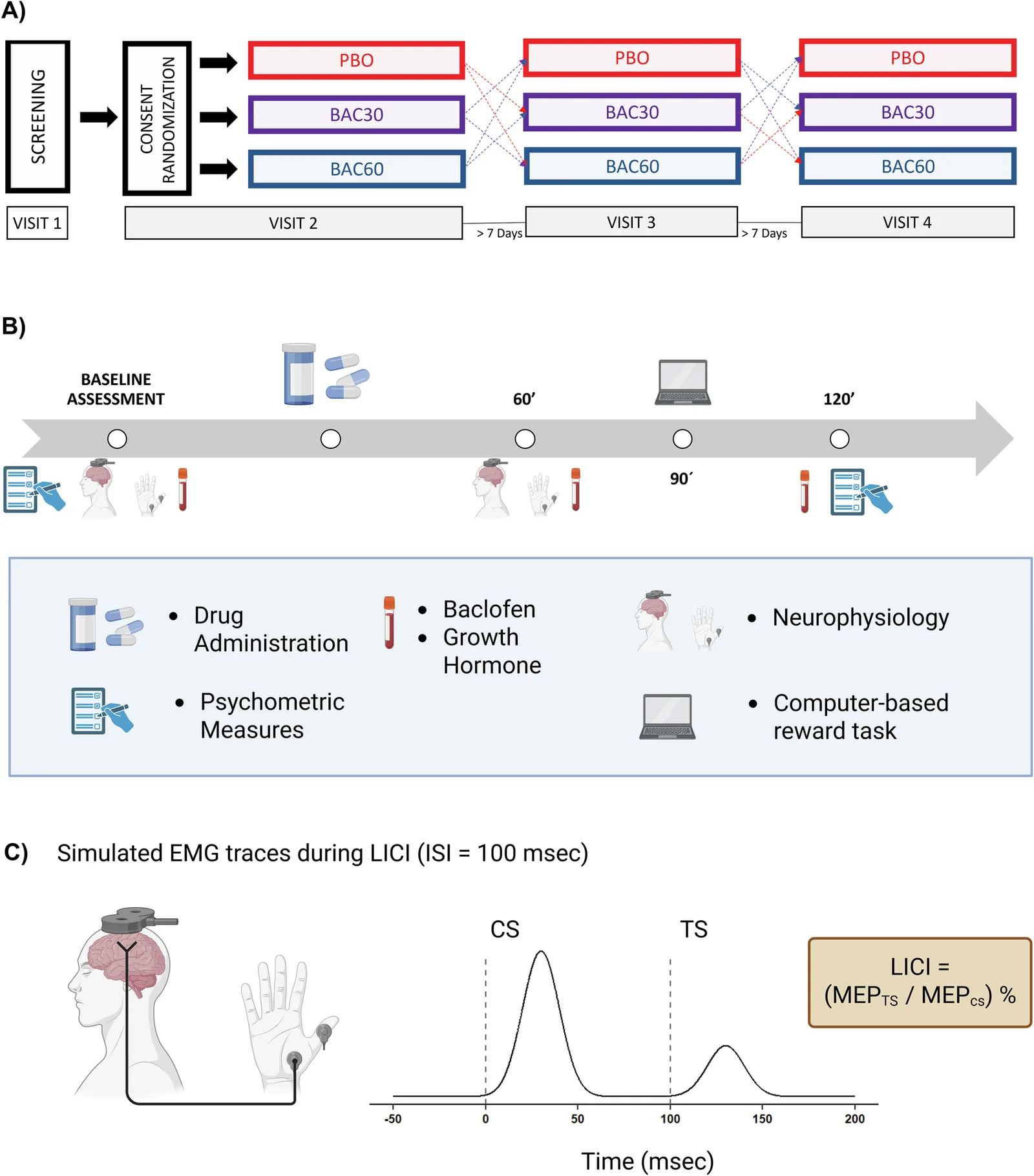
Study design, visit timeline, and primary outcome. **A** Overview of the randomized, double-blind, placebo-controlled crossover design. **B** Timeline of procedures during each experimental visit. **C** Primary outcome measure. BAC30, baclofen 30 mg; BAC60, baclofen 60 mg; PBO, placebo.

This was a randomized, double-blind, placebo-controlled, crossover experimental medicine study in healthy volunteers. Each participant completed three experimental sessions separated by ≥7 days. Participants received baclofen 30 mg (BAC30), 60 mg (BAC60), or matching placebo (PBO) in a counterbalanced Williams square design, stratified by sex. Participants, investigators, and outcome assessors were blinded to treatment allocation. Randomization codes were held by the hospital pharmacy and accessed only in case of medical emergency. The schedule of events for each session is provided in Fig. 1). All methods were performed in accordance with the relevant guidelines and regulations. The study was granted an exemption by the Swedish Medical Products Agency (MPA), and was approved by the Swedish Ethics Review Authority (2023-00556-01). All participants provided written informed consent. Study procedures and analysis plan were preregistered at https://osf.io/scdqu/overview.

### Participants

Thirty-two non-treatment-seeking healthy volunteers were recruited: 16 HD and 16 LD, with each group balanced for sex. Participants were recruited as non-treatment-seeking HD and LD, consistent with experimental medicine models designed to investigate alcohol-related neurobiological mechanisms while minimizing confounding from psychiatric comorbidity and pharmacological treatment. Drinking groups were defined according to sex-specific Swedish national risk-drinking recommendations (≥14 standard drinks per week for males and ≥9 standard drinks per week for females), which closely correspond to sex-specific NIAAA risk thresholds [27]. Participants were classified as LD if they were healthy social drinkers whose regular alcohol consumption remained below the corresponding sex-specific risk-drinking thresholds. All LD reported current alcohol consumption, although intake was low in some participants; no abstainers were included. Participants were classified as HD if they met the sex-specific weekly drinking threshold over the preceding four weeks. Participants whose average weekly consumption fell below these thresholds were also classified as HD if they showed other indicators of risky drinking, including recurrent binge-drinking episodes (>1 episode/month in the last six month) and/or AUDIT scores above the established screening cutoff (see Table S1).

All participants were aged 20–65 years, fluent in Swedish, and in good general health. Female participants underwent urine pregnancy testing, and all participants underwent drug and alcohol testing at screening and before each session. Participants were also instructed to avoid non-essential medications, including NSAIDs and antibiotics, during the days preceding study visits, and to standardize caffeine and nicotine intake by consuming their last coffee and cigarette approximately two hours before baseline assessments. Exclusion criteria included clinically significant medical illness, current moderate or severe DSM-5 psychiatric disorders (including moderate-to-severe alcohol or other substance use disorders), pregnancy, contraindications to baclofen or TMS [28], or use of contraindicated medications. Psychiatric screening was performed using the Modified Mini Screen (MMS, [29]), with follow-up MINI interviews conducted when indicated. Only participants without a current psychiatric diagnosis were enrolled. No participants were taking psychotropic medications or other medications known to affect GABAergic neurotransmission or cortical excitability during the study. Stable hormonal contraceptive use was permitted in female participants. Participants were withdrawn from the study upon request, in the event of serious adverse events, pregnancy, protocol non-compliance, or at the discretion of the principal investigator judgement.

### GABA_B_ receptor manipulation: baclofen administration

Baclofen was administered orally at single doses of 30 mg and 60 mg. Dose selection was informed by previous human pharmacodynamic studies showing that 10 mg produced minimal GH responses in healthy volunteers, whereas 60 mg elicited robust GABA_B_-mediated endocrine effects [23]. Higher doses (90 mg) have been investigated in patients with alcohol use disorder [30] but were not considered appropriate in the present study because our participants did not meet diagnostic criteria for AUD and were therefore expected to have lower drug tolerance. The use of identical doses in both groups also enabled direct comparison of pharmacodynamic responses under equivalent systemic exposure. Baclofen reaches peak plasma concentrations approximately 30–90 min after oral administration, and has an elimination half-life of approximately 3–4 h. Active drug and placebo were prepared by over-encapsulating 20 mg baclofen tablets in opaque gelatin capsules. Placebo capsules contained inert filler and were visually identical. Capsules were prepared under Good Manufacturing Practice (GMP) conditions. Blinding and randomization were by a pharmacy contractor, insulated from the study team. Drug accountability was maintained throughout the study. Independent quality monitoring was by Forum Östergötland, a node of Clinical Trials Sweden. Treatment codes were only delivered to the study team once monitoring was completed and data were locked.

### Neurophysiological assessment

TMS–EMG assessments were performed with participants seated comfortably at rest. EMG activity was recorded from the right abductor pollicis brevis (APB) muscle using silver–silver chloride surface electrodes in a muscle-belly configuration, with a wrist ground electrode. EMG signals were sampled at 10 kHz, band-pass filtered between 20 and 2500 Hz, and recorded in 200-ms epochs time-locked to the TMS pulse.

Monophasic TMS pulses were delivered in single and paired-pulse protocols using a figure-of-eight coil connected to a Magstim BiStim stimulator. The motor hotspot for the APB was identified and marked for consistency. Resting motor threshold (RMT) was defined as the minimum intensity eliciting MEPs ≥50 μV in 5 of 10 trials. LICI was assessed using paired suprathreshold pulses (130% RMT) at a 100 ms interstimulus interval (Fig. 1C). SICI was assessed using a subthreshold conditioning pulse (80% RMT) followed by a suprathreshold test pulse (130% RMT) at a 2 ms interval. ICF was assessed using the same intensities at a 20 ms interval. Ten trials were recorded per condition. Test MEP amplitudes were expressed as a percentage of the unconditioned test pulse. Assessments were performed at baseline and ~60 min after drug administration.

### Blood biomarkers

At the beginning of each session, an indwelling intravenous catheter was placed for serial blood sampling. Blood samples were collected at predefined time points for the assessment of plasma baclofen concentrations and growth hormone (GH) levels. Due to technical difficulties during venipuncture (e.g., low blood pressure or participant discomfort) or insufficient sample quality, not all planned blood samples could be obtained or analyzed. In total, 276 GH samples and 268 plasma baclofen samples were available for analysis. For baclofen, two samples were missing among LD at 2 h post-administration during the PBO (n = 1) and BAC60 (n = 1) sessions, respectively. Among HD, plasma samples were unavailable from one female participant at all time points, and an additional six samples were missing at 2 h post-administration across different sessions (PBO: *n* = 2; BAC30: *n* = 3; BAC60: *n* = 1).

Baclofen was quantified by liquid chromatography with tandem mass spectrometry (LC-MS/MS). In brief, blood samples were prepared by protein precipitation with 0.075% formic acid in acetonitrile/ethanol 90/10. After mixing and centrifugation, 3 µL of the clear supernatant was injected. Sample analysis was performed using an Agilent 1290 Infinity UHPLC system coupled to an Agilent 6460 triple quadrupole (Santa Clara, CA, USA). The calibration curve was linear over 0.005 – 2.0 μg/mL. The limit of quantification (LOQ) was 0.005 μg/mL.

Plasma GH levels were measured using an electrochemiluminescence immunometric assay (ECLIA) on a Cobas Analyzer (Roche Diagnostics) with a sensitivity of 0.05 μg/L. Analyses were carried out by the accredited Clinical Biochemistry Department of Linköping University Hospital.

### Statistical analysis

The sample size was determined a priori using a power analysis based on the expected effect on the primary neurophysiological outcome, LICI, as specified in the preregistered study protocol. Previous work reported a large effect of baclofen on LICI in healthy volunteers (Cohen’s d = 1.1; [18]) and large group differences in LICI between individuals with severe AUD and healthy controls (Cohen’s d > 2; [31]). To adopt a more conservative estimate, the study was powered to detect an effect size of 0.8 with 80% power at α = 0.05.

Analyses were conducted in R (v4.2.3). Statistical significance was set at α = 0.05 (two-tailed). Custom R scripts used for data processing, statistical analyses, and figure generation are available from the corresponding author upon reasonable request. Repeated-measures ANOVA effect sizes are reported as partial eta squared (η²); linear mixed-effects model estimates are reported as unstandardized regression coefficients (β) with Satterthwaite-adjusted degrees of freedom.

Group differences in demographic and psychometric variables were assessed using independent-samples t-tests or χ² tests. Baseline cortical excitability was analyzed using repeated-measures ANOVA (rmANOVA) with treatment as a within-subject factor and group as a between-subject factor.

Baclofen blood concentrations and plasma GH levels were analyzed using rmANOVAs with treatment and time as within-subject factors and group as a between-subject factor, applying Greenhouse–Geisser corrections where appropriate.

Neurophysiological parameters of interest, such as LICI, SICI, and ICF were analyzed using three-way rmANOVAs with treatment and time as within-subject factors and group as a between-subject factor.

In accordance with the preregistered analysis plan, sex was initially included as a between-subject factor in the rmANOVA examining baclofen effects on LICI, but was not retained because it did not significantly contribute to the model. Similarly, visit order was initially included, but not retained because of lack of effect. This strategy was preregistered and aimed to avoid unnecessary model complexity while preserving sensitivity to potentially meaningful sex and order effects.

Because of individual variability in baseline, in addition to the preregistered analysis, we conducted a repeated-measures analysis of covariance (rmANCOVA) on the absolute change in LICI (ΔLICI = LICI_1h_ – LICI_baseline_). Absolute change was preferred over relative change to preserve the measurement scale and avoid covariation of numerator and denominator. Baseline LICI was included as a covariate to control for baseline-dependent variability.

All post-hoc analyses were conducted using estimated marginal means. All contrasts were Benjamini–Hochberg (BH)-adjusted for multiple comparisons.

## Results

### Participant characteristics

**Fig. 2:**
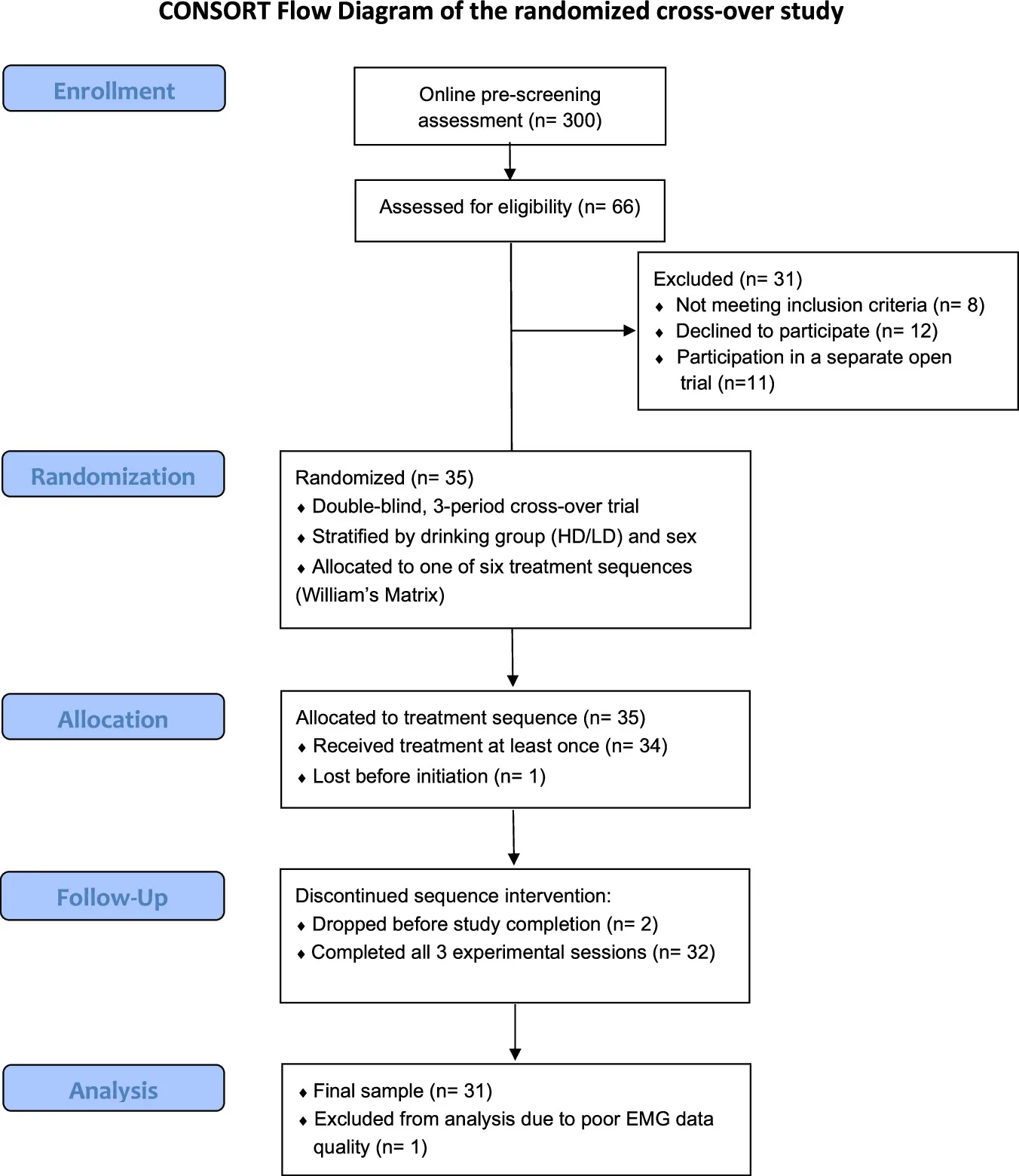
CONSORT Flow Diagram for Randomized Crossover Study Design. The diagram summarizes participant enrolment, treatment allocation, follow-up, and inclusion in the analysis.

Participant disposition is shown in Fig. 2. The final analysis sample included 16 drinkers (HD; 8 females, 8 males) and 15 light drinkers (LD; 7 females, 8 males). HD and LD did not differ in age, sex and hormonal therapy distribution or handedness (all *p* > 0.05, see Table 1). By design, they differed in their number of drinks per week (LD: 4.39 ± 3.54, HD: 14.91 ± 6.86; *p* < 0.001). Because heavy drinking was defined using sex-specific thresholds (≥14 drinks/week for men and ≥9 drinks/week for women), women in the HD group reported a mean alcohol consumption of 13.2 ± 4.4 drinks/week compared with 2.8 ± 1.6 drinks/week in LD women, whereas men reported 16.6 ± 8.7 versus 5.8 ± 4.2 drinks/week, respectively (sex-stratified baseline characteristics are additionally reported in Table S1). As expected, HD also had higher AUDIT scores than LD (LD: 5.2 ± 2.4, HD: 14.0 ± 4.8; *p* < 0.001; Table 1**)**.

**Table 1:** Baseline demographic and clinical characteristics of light drinkers (LD) and heavy drinkers (HD).

	Light Drinkers (*n*: 15)	Heavy Drinkers (*n*:16)	p
**Female**, *n* (%)	7 (46.7)	8 (50.0)	1.000
**Hormonal therapy**, *n* (%)	3 (20.0)	5 (31.2)	0.761
**Age**	28.03 (8.87)	24.04 (4.58)	0.125
**Left Handedness**, *n* (%)	0 (0.0)	2 (12.5)	0.494
**EHI**	7.20 (1.15)	5.38 (4.63)	0.149
**DUDIT**	0.13 (0.52)	1.06 (1.95)	0.084
**AUDIT**	5.20 (2.40)	14.00 (4.80)	**<0.001**
**Average n. drinks/week**	4.39 (3.54)	14.91 (6.86)	**<0.001**
**Binge Drinking Episodes**, *n* (%)			**<0.001**
→ Almost every day	0 (0)	1 (6.2)	
→ Once per week	0 (0)	12 (75)	
→ Once per month	5 (33.3)	3 (18.8)	
→ Less than once per month	8 (53.3)	0 (0)	
→ Never	2 (13.3)	0 (0)	
**MADRS**	4.20 (3.36)	6.00 (3.93)	0.183

Values are presented as mean (SD) unless otherwise indicated. Group comparisons were performed using independent-samples t tests for continuous variables and Fisher’s exact test for categorical variables. LD and HD groups did not differ significantly in age, sex distribution and handedness measures. As expected, HD reported higher AUDIT scores and greater average weekly alcohol consumption compared with LD (p < 0.001).

*AUDIT* alcohol use disorders identification test, *DUDIT* drug use disorders identification test, *EHI* edinburgh handedness inventory, *NEO* revised NEO personality inventory.

The HD group showed higher resting motor thresholds (RMT) prior to drug administration than LD (*F*(1,29) = 7.85, *p* = 0.009). RMT also showed a trend toward within-subject differences across sessions (*p* = 0.052), but there was no grou*p* × session interaction (*p* = 0.566). For all other measures of cortical excitability—intracortical facilitation (ICF), long-interval intracortical inhibition (LICI), and short-interval intracortical inhibition (SICI)—there were no main effect of group or session, and no grou*p* × session interactions (all *p* > 0.15). Thus, overall, with the exception of RMT, baseline cortical excitability did not differ between HD and LD, nor did it vary systematically across measurement sessions (see Table 2).

**Table 2:** Cortical excitability measures in light drinkers (LD) and heavy drinkers (HD) across placebo, baclofen 30 mg, and baclofen 60 mg conditions.

	Light Drinkers			Heavy Drinkers		
	PBO	BAC30	BAC60	PBO	BAC30	BAC60
**RMT**						
Baseline	40.82 (5.17)	45.67 (5.63)	40.26 (5.50)	41.38 (5.53)	46.97 (5.92)	45.99 (5.48)
1 h	40.72 (5.33)	40.92 (5.75)	40.41 (5.13)	45.58 (6.46)	46.97 (5.05)	46.68 (6.11)
**MEP amplitude for SP 130%**						
Baseline	361.22 (363.25)	267.23 (168.12)	194.68 (103.17)	180.91 (102.71)	168.84 (92.89)	164.06 (81.59)
1 h	296.89 (227.73)	285.99 (174.79)	217.45 (91.81)	198.45 (99.19)	184.92 (95.27)	131.87 (52.39)
**LICI**						
Baseline	24.39 (12.61)	28.28 (16.89)	26.58 (12.86)	28.71 (16.98)	31.96 (15.48)	34.72 (22.62)
1 h	25.86 (14.75)	18.31 (10.26)	16.92 (7.53)	25.24 (14.45)	25.22 (14.50)	35.94 (21.33)
**SICI**						
Baseline	48.02 (30.10)	34.74 (18.25)	37.72 (24.44)	38.44 (25.02)	44.57 (25.21)	58.61 (54.25)
1 h	45.84 (27.03)	41.21 (28.32)	33.35 (22.38)	37.80 (23.05)	61.56 (41.06)	51.57 (33.71)
**ICF**						
Baseline	103.28 (42.41)	113.93 (29.67)	124.40 (67.69)	84.35 (40.65)	96.12 (66.83)	101.86 (56.59)
1 h	145.25 (54.58)	95.79 (27.48)	85.83 (38.28)	93.61 (51.54)	93.91 (52.73)	112.39 (41.37)

Values represent mean (SD). Measures of corticospinal excitability (resting motor threshold [RMT] and single-pulse MEP amplitude), GABAB-mediated inhibition (LICI), GABAA-mediated inhibition (SICI), and intracortical facilitation (ICF) were collected at baseline and 1 h after drug administration, corresponding to peak baclofen exposure. Across groups, placebo produced minimal changes in cortical excitability. Baclofen produced dose-dependent reductions in ICF and LICI in LD, consistent with enhanced GABAB-mediated inhibition, whereas HD showed attenuated or inconsistent modulation across doses. SICI exhibited no systematic drug-related changes in either group.

*RMT* resting motor threshold, *MEP* motor-evoked potential, *LICI* long-interval intracortical inhibition, *SICI* short-interval intracortical inhibition, *ICF* intracortical facilitation, *PBO* placebo, *BAC30* baclofen 30 mg, *BAC60* baclofen 60 mg.

### Baclofen blood levels

**Fig. 3:**
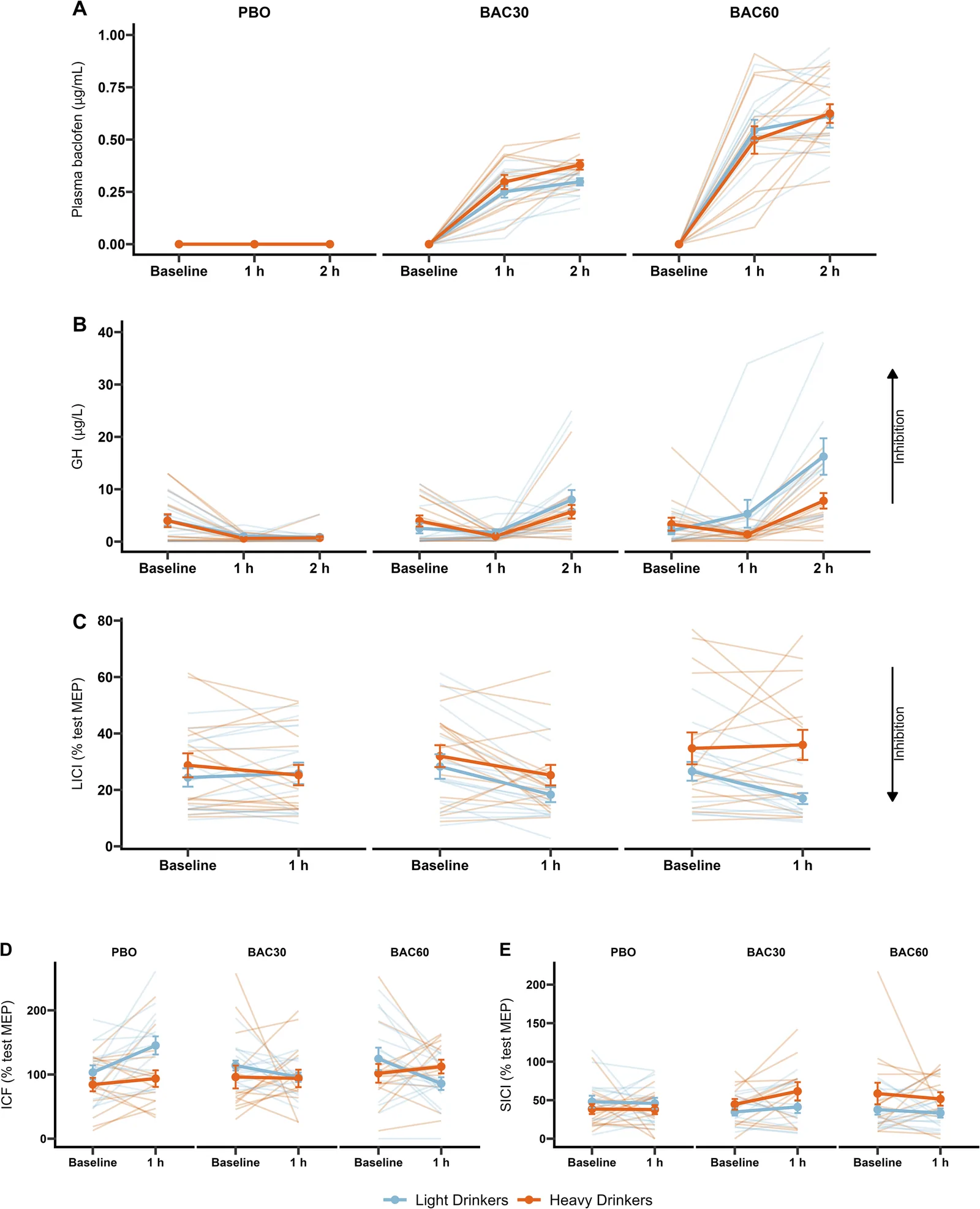
Pharmacokinetic, endocrine, and neurophysiological responses to baclofen by drinking group. **A** Mean ( ± SE) blood baclofen concentrations at baseline, 1 h, and 2 h following placebo, baclofen 30 mg, and baclofen 60 mg administration. **B** Mean ( ± SE) plasma growth hormone (GH) concentrations measured at the same time points. **C** Mean ( ± SE) long-interval intracortical inhibition (LICI), expressed as conditioned MEP amplitude (% of test stimulus), measured before and 1 h after each treatment. **D** Mean ( ± SE) intracortical facilitation (ICF). **E** Mean ( ± SE) short-interval intracortical inhibition (SICI).Thin lines represent individual participant trajectories, whereas thick lines indicate group-level means. Blue lines represent light drinkers and orange lines HD.

To confirm exposure and account for potential individual variation in pharmacokinetics, baclofen blood concentrations were measured 1 and 2 h after drug intake (Table S3, Fig. 3a), and analyzed with *treatment* (placebo, baclofen 30 mg, baclofen 60 mg) and *time* (baseline, 1 h, and 2 h post-drug)) as within-subject factors in an rmANOVA model that included 14 HD and 14 LD individuals due to some missing samples.

We found the expected main effects of *treatment* (*F*(1.22, 25.55) = 127.42, *p* < 0.001, η²=0.82) and *time* (*F*(1.68, 35.30) = 151.16, *p* < 0.001, η² = 0.86), as well as a robust *treatment* × *time* interaction (*F*(2.49, 52.24) = 73.09, *p* < 0.001, η² = 0.76; Table S5). No main or interaction effects of group were detected (all *p* > 0.10), indicating comparable baclofen exposure between LD and HD. These findings confirm an adequate, dose-dependent systemic exposure to baclofen, and establish a lack of group-related pharmacokinetic differences that could otherwise confound the functional measures. Blood levels were subsequently included as covariates in mixed-effects models to account for individual differences in pharmacokinetics.

### Growth hormone (GH) response

Because GH secretion is known to depend on activations of GABA_B_Rs in the pituitary gland, we next examined GH release 1 and 2 h after drug intake as a neuroendocrine marker of receptor engagement (Table S4, Fig. 3b). A two-way repeated-measures ANOVA, including 15 HD and 12 LD individuals revealed significant main effects of *treatment* (*F*(1.31, 32.6) = 17.5, *p* < 0.001, η² = 0.41) and *time* (*F*(1.31, 32.6) = 16.6, *p* < 0.001, η² = 0.40; Table S6).

There was a robust *treatment* × *time* interaction (*F*(2.6, 65.9) = 14.6, *p* < 0.001, η²ₚ = 0.37), confirming that GH release was both dose- and time-dependent. Also, *group* × *time* (*F*(1.29, 32.4) = 4.0, *p* = 0.04, η² = 0.15) and *group x treatment* (F(1.31, 32.6) = 4.1, *p* = 0.04, η² = 0.14) interactions suggested a possible difference in the temporal dynamics of GH release, and a difference in baclofen sensitivity between groups, despite comparable overall response magnitude. No *group x drug x time* interaction was observed.

*Post hoc* analyses provided further detail on dose- and time-dependent GH dynamics. Under placebo, GH declined over time in HD (baseline vs 1 h: *p* = 0.007; baseline vs 2 h: *p* = 0.011) but remained stable in LD. Following BAC30, HD showed an early reduction in GH (baseline vs 1 h: *p* = 0.007) followed by a rebound from 1 h–2 h (*p* = 0.026), whereas LD exhibited a delayed increase at 2 h (*p* = 0.031). At BAC60, GH increased robustly after 2 h, particularly in LD (baseline vs 2 h and 1 h vs 2 h: both *p* < 0.001), while HD showed a significant rise only between 1 h and 2 h (*p* = 0.007). A trend toward higher GH levels in LD versus HD emerged at 2 h following 60 mg (*p* = 0.07). For additional details, see Supplementary Results.

Together with the pharmacokinetic results, these findings demonstrate effective baclofen exposure and baclofen-induced neuroendocrine GABA_B_Rs response, although this response showed an altered time course in HD.

### Effects of baclofen on long-interval intracortical inhibition

In line with our preregistered hypotheses, we conducted an rmANOVA with treatment and timepoint as within-subject factors, and group as a between-subject factor (Table S7, Fig. 3c). The analysis revealed a main effect of *timepoint* (*F*(1,29) = 11.65, *p* = 0.002, η²=0.29), indicating an overall change in cortical inhibition from baseline to 1 h after drug administration. A *group* × *treatment* interaction (*F*(1.7, 49.4) = 3.68, *p* = 0.031, η²=0.11) indicated that the effects of baclofen on LICI differed between LD and HD participants. In addition, there was a trend for a *treatment* × *timepoint* interaction (*F*(1.95, 56.4) = 2.92, *p* = 0.062, η² = 0.09). We also observed a three-way interaction (*group* × *treatment* × *timepoint*; *F*(1.95,54.4) = 3.36, *p* = 0.042, η² = 0.10), indicating that treatment-related changes over time differed between groups.

*Post-hoc* analyses revealed that BAC30 significantly increased LICI in both LD and HD (*p* = 0.005 and 0.043, respectively). In contrast, BAC60 increased LICI in LD (*p* = 0.014) but not in HD (*p* = 0.73), resulting in a significant group difference at the high dose (*p* = 0.003). For additional details, see Supplementary Results.

Sex did not interact with treatment or group (all *p* > 0.05), although its inclusion modestly reduced residual variance (Table S8). Including treatment order in the model also did not reveal any main or interaction effects (all *p* > 0.10). Importantly, the grou*p* × treatment interaction remained significant, and the grou*p* × treatment × timepoint interaction remained at trend level, indicating that the differential baclofen response between HD and LD was independent of treatment order (Table S9). Adjustment for individual plasma baclofen concentration did not materially alter the group-dependent treatment effect on LICI. Plasma baclofen concentration itself was not associated with LICI (F(1137.21) = 0.007, *p* = 0.934), whereas the treatment × group interaction remained significant (F(2128.88) = 3.84, *p* = 0.024, η²*p* = 0.06) (Table S10).

**Fig. 4:**
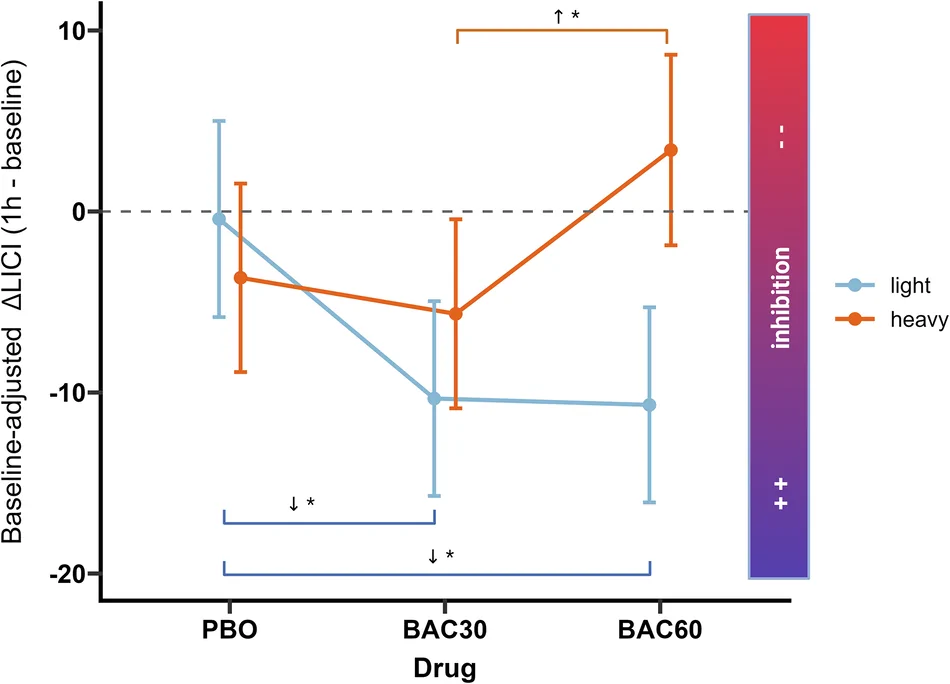
Absolute change in long-interval intracortical inhibition (ΔLICI = 1 h − baseline) by drug and drinking group. Points represent individual participants; lines and error bars depict group-level estimated marginal means ± 95% confidence intervals derived from the baseline-adjusted repeated-measures ANCOVA model. Negative values represent stronger inhibitory tone (decreased LICI), The dashed horizontal line indicates no change from baseline.

Because baseline LICI responses showed considerable individual variation, in addition to the preregistered analyses, we carried out an rmANCOVA to evaluate the effects of baclofen on the absolute change in LICI (ΔLICI) by drinking group, controlling for baseline inhibitory tone (baseline LICI) (Fig. 4, Table S11). No main effect of drinking group was observed on ΔLICI (*F*(1, 28) = 1.99, *p* = 0.17), while a main effect of drug was observed (*F*(2, 57) = 3.94, *p* = 0.025, η²=0.12), demonstrating that ΔLICI was influenced by treatment. The drug × group interaction was also significant (*F*(2, 57) = 6.17, *p* = 0.004, η² = 0.18), again indicating differential effects of baclofen on cortical inhibition between HD and LD. *Post hoc* comparisons showed no significant LD–HD differences under placebo or BAC30. In contrast, under BAC60, LD exhibited a markedly greater reduction in LICI compared with HD (LD: −10.69 [ − 16.08, −5.30] vs HD: +3.39 [ − 1.87, 8.66]), suggesting an attenuated response to high-dose baclofen in HD. For details, see Supplementary Results.

Morever, when included as a covariate, baseline LICI explained a significant proportion of variance in ΔLICI at both the between-subject level (*F*(1, 28) = 15.44, *p* = 0.0005, η² = 0.36) and the within-subject level (*F*(1, 57) = 20.66, *p* < 0.001, η² = 0.27). This shows that baseline inhibitory tone substantially influenced subsequent baclofen-induced modulation of cortical inhibition. Importantly, the drug × group interaction remained significant after additionally adjusting for session-specific baseline RMT (F(2,57.60) = 5.27, *p* = 0.008, η²*p* = 0.15), while RMT itself was not associated with ΔLICI (*p* = 0.348; Table S11). Thus, although the preregistered analytical model and the rmANCOVA showed similar results, the latter model showed higher sensitivity, and detected an influence of baseline LICI on the baclofen responses. This model is therefore likely preferable in future studies.

In an exploratory analysis, instead of the LD – HD categorization, we examined whether AUDIT scores or weekly alcohol consumption were continuous moderators of the effect exerted by baclofen on ΔLICI. Neither AUDIT score (drug × AUDIT: F(2,57.64) = 1.96, *p* = 0.151) nor weekly alcohol consumption (drug × drinks/week: F(2,57.61) = 1.10, *p* = 0.338) significantly moderated the pharmacodynamic response to baclofen. Similarly, baseline LICI was not linearly associated with AUDIT score (F(1,29) = 0.55, *p* = 0.466; Table S12).

### Secondary cortical excitability measures

To assess whether baclofen also affected corticospinal excitability or other intracortical processes, we examined RMT, SICI and ICF using analogous rmANOVAs (Table S13). For RMT, a significant main effect of group was observed (*F*(1,29) = 8.31, *p* = 0.007, η² = 0.22), with HD exhibiting higher thresholds than LD. No main effects of treatment or timepoint, nor any interaction effects, were detected (all *p* > 0.10), indicating stable group differences in corticospinal excitability that were not modulated by baclofen. For ICF, a significant treatment × timepoint interaction was observed (*F*(2,46) = 4.52, *p* = 0.016), indicating that intracortical facilitation changed over time as a function of treatment (Fig. 3d). However, this effect did not differ between LD and HD, and no group-related interactions were detected (all *p* > 0.14). For SICI, there were no significant main effects of group or treatment, nor any treatment × timepoint or higher-order interactions (all *p* > 0.12). Trend-level effects of timepoint and grou*p* × timepoint did not survive correction and were not modulated by treatment, indicating the absence of a specific pharmacological effect on SICI (Fig. 3e).

Together, these findings indicate that baclofen’s neurophysiological effects were selective for GABA_B_R-mediated cortical inhibition, with no consistent modulation of GABA_A_R-dependent inhibition or intracortical facilitation.

## Discussion

We used TMS to probe GABA_B_R–mediated cortical inhibition following acute baclofen administration in individuals with different levels of alcohol consumption. Consistent with prior studies [18, 20], baclofen potentiated LICI across participants, but this effect did not show simple dose-dependence. In LD, both 30 mg and 60 mg increased inhibition to a comparable extent, suggesting that potentiation of LICI may have approached a plateau within this dose range. In HD, 30 mg potentiated LICI with a magnitude similar to that seen in LD, whereas 60 mg failed to produce further potentiation, resulting in a divergence between groups under the higher-dose condition.

Our observation that potentiation of LICI by baclofen reached a plateau at 30 mg in LD and did not increase further at 60 mg has potentially important implications. Previous human pharmacological TMS data are limited; however, McDonnell et al. reported a similar enhancement of LICI following a single 50 mg dose in healthy volunteers [18]. Together, these findings raise the possibility that LICI reaches a physiological plateau within the range of baclofen doses that can be safely administered acutely to healthy individuals. Whether higher doses would produce additional modulation, or whether LICI represents a saturable pharmacodynamic measure remains unknown.

Our findings that the pattern of LICI potentiation across baclofen doses observed in HD differed from that in LD are generally in line with prior observations that a history of alcohol exposure can influence GABA_B_-mediated responses. However, changes in sensitivity to GABAergic agents, including baclofen, following a history of alcohol exposure are complex. In preclinical models, both baclofen and the GABA_B_ positive allosteric modulator ADX71441 suppress alcohol self-administration with a dose-response curve that is left-shifted compared to non-dependent animals, indicating increased sensitivity after chronic exposure [14, 32]. In contrast, AUD patients often tolerate much higher baclofen doses than other patients [15].

In line with the latter observation, Durant et al. reported blunted effects at high baclofen doses (60 and 90 mg) in abstinent individuals with AUD [23], despite comparable baclofen pharmacokinetics, suggesting that the reduced pharmacodynamic response was unlikely to be explained by differences in systemic drug exposure. In agreement with those findings, our study demonstrates that group differences emerge predominantly under higher levels of agonist stimulation. Similarly, plasma baclofen concentrations did not differ between HD and LD, supporting the interpretation that the observed differences in the ability of baclofen to potentiate cortical inhibition primarily reflect altered neurophysiological responsiveness rather than altered pharmacokinetics. The neural mechanism underlying an escape of baclofen-induced LICI potentiation in HD remains unknown, but given the preserved response at the lower dose, it is unlikely to reflect a uniformly attenuated response to GABA_B_Rs activation.

The attenuated response to BAC60 in HD remained after adjustment for baseline inhibition, indicating that it cannot be explained by differences in resting inhibitory tone. Moreover, LICI did not differ between groups at baseline, contrasting with reports of reduced baseline LICI in male patients with severe AUD [31]. Similarly, in contrast to previous neuroendocrine challenge studies [22, 24, 30, 33], baclofen elicited a robust GH response in both HD and LD, with no between-group differences despite a modest attenuation and delay following BAC60 in HD.

Apparent discrepancies between our data and prior findings should be interpreted in the context of the populations investigated. Previous studies examined older, exclusively male patients with established AUD, often during abstinence following detoxification [30, 31]. In contrast, our participants were young, sex-balanced, non-treatment-seeking heavy drinkers who did not meet criteria for AUD, had never experienced clinically significant alcohol withdrawal, and were all able to abstain from alcohol for at least 72 h before each experimental session without developing withdrawal symptoms. Together, these findings suggest that alterations in cortical GABA_B_Rs-mediated responsiveness may emerge earlier than tonic inhibitory deficits or endocrine abnormalities during the progression from sustained heavy drinking to alcohol use disorder. Although our sample was sex-balanced and we did not observe sex-driven effects, hormonal influences on GABAergic transmission [34] warrant investigation in larger cohorts including both female and male participants.

Few studies have examined neurophysiological correlates of alcohol exposure in non-clinical populations spanning different levels of drinking. Among these, Kaarre et al. investigated young adults with a long history of heavy alcohol use compared with controls reporting little or no alcohol use, and found a dimensional association between AUDIT-C scores and baseline frontal N45 amplitude [35]. In the present study, exploratory dimensional analyses did not identify a significant continuous association between AUDIT score and baseline LICI, nor did AUDIT score significantly moderate baclofen-induced ΔLICI. However, these analyses interrogate different neurophysiological phenomena: N45 represents a baseline TMS-EEG measure of GABAergic activity, whereas ΔLICI reflects the acute pharmacodynamic response to GABA_B_Rs activation. Moreover, given the observational nature of alcohol exposure in the present study, ΔLICI should not be interpreted as a quantitative marker of alcohol-related damage. Whether GABA_B_-mediated cortical responsiveness varies dimensionally with the amount, duration, or severity of alcohol exposure remains to be established.

Notably, Kaarre et al. also found unchanged RMT in their sample [35]. Previous findings on RMT in relation to alcohol exposure have been heterogeneous, including unchanged or reduced RMT across different populations [21]. In our study RMT was assessed at each of three sessions and showed no visit × group interaction, suggesting a stable group difference. Higher RMT in HD may also appear counterintuitive in the context of the hyperexcitable state classically associated with chronic alcohol exposure. However, allostatic neuroadaptation is unlikely to represent a uniform increase in cortical excitability. Rather, chronic alcohol exposure produces an imbalance between excitatory and inhibitory systems, and increased glutamatergic drive may coexist with inefficient or dysregulated cortical network function [36]. Higher RMT in HD may therefore reflect this broader alteration in excitation–inhibition balance rather than contradicting an allostatic model of alcohol-related neuroadaptation.

A key strenght of the present design is the simultaneous assessment of endocrine and cortical biomarkers indexing partially distinct pharmacodynamic mechanisms, potentially produced within different compartments of drug exposure. GH and LICI are not interchangeable measures of central pharmacodynamic activity. Baclofen-induced GH release reflects activation of the hypothalamic-pituitary neuroendocrine axis, which is directly exposed to circulating drug concentrations [37], whereas LICI reflects GABA_B_Rs-mediated neurotransmission within a central compartment protected by the blood-brain barrier [38].

Moreover, although both LICI and GH responses ultimately depend on GABA_B_Rs activation, they interrogate different physiological compartments and different aspects of GABA_B_R signaling. One plausible interpretation is that GH release predominantly reflects modulation of presynaptic neuroendocrine pathways regulating hypothalamic hormone secretion [39], whereas LICI primarily reflects slow postsynaptic GABA_B_Rs-mediated inhibition in cortical pyramidal neurons [18, 20]. Accordingly, preservation of GH responses does not necessarily imply preservation of cortical pharmacodynamic responsiveness.

Consistent with this framework, plasma baclofen concentrations were comparable between HD and LD, GH responses remained largely preserved, but cortical LICI responses diverged substantially following BAC60. This progressive dissociation suggests that mechanisms beyond systemic drug exposure contribute to altered cortical GABA_B_Rs responsiveness in HD. The present study cannot determine the mechanisms underlying this dissociation. Possible explanations include altered receptor sensitivity, changes in downstream intracellular signaling, or broader neuroadaptive changes affecting cortical inhibitory networks after sustained heavy alcohol exposure.

We cannot exclude differences in compartment-specific drug distribution due to altered blood-brain-barrier (BBB) permeability in HD. Whether this is altered in non-dependent heavy drinkers is presently unknown. Limited older evidence suggests that greater alcohol consumption may be associated with increased blood–CSF barrier permeability, while acute alcohol administration at moderate intoxicating concentrations does not measurably increase circulating S100B. Direct studies using contemporary measures of BBB permeability in well-characterized non-dependent heavy drinkers appear to be lacking. Of note, an increased BBB permeability in HD would be expected to result in increased central baclofen exposure, and thus is unlikely to account for the absence of a baclofen response at 60 mg.

The absence of SICI modulation supports that the observed effects are selective for GABA_B_R-mediated inhibitory processes, and argue against a generalized shift in inhibitory tone. While previous findings support altered GABA_B_R-mediated inhibition in both motor cortex and prefrontal cortical regions of recovering AUD patients [21], present results suggest that this dysregulation may be detectable even before clinical AUD develops, in particular under high receptor activation.

Our study has limitations. The sample size, although adequately powered for primary effects, limits detection of small effect sizes or higher-order interactions. Nevertheless, the robust detection of group-dependent effects on LICI, contrasted with the absence of comparable effects on GH, provides useful information regarding the relative sensitivity of these complementary pharmacodynamic biomarkers for assessing GABA_B_R target engagement. Baseline variability in cortical excitability is well recognized in paired-pulse TMS studies and likely reflects the combined influence of biological state variables—including sleep, stress, mood, circadian factors and recent substance exposure—as well as intrinsic measurement variability. Although several potential confounders were minimized through standardized testing procedures, residual variability is unavoidable and motivated the complementary baseline-adjusted analysis. Also, heavy drinking was defined based on recent (6-month) consumption, and lifetime exposure was not systematically quantified. Furthermore, the study deliberately enrolled non-treatment-seeking heavy drinkers without current psychiatric disorders rather than patients with clinical AUD. Therefore, these findings may not directly generalize to clinical AUD populations and should be interpreted as reflecting neurophysiological alterations associated with sustained heavy alcohol consumption. The cross-sectional design precludes causal inference, and limits our ability to determine whether altered GABA_B_Rs responsiveness reflects alcohol effects, compensatory neuroadaptations, or pre-existing vulnerability. Longitudinal studies incorporating periods of monitored abstinence will have to clarify whether these alterations normalize over time and whether they precede, accompany, or follow escalation of drinking.

Future studies should also test whether GABA_B_R PAMs normalize LICI responses in HD and whether baseline LICI predicts clinical or behavioral response in trials with longer follow-up. Collectively, our findings position LICI as a promising, non-invasive biomarker of GABA_B_R target engagement in a central compartment, and suggest that altered cortical pharmacodynamic responsiveness under higher agonist drive may represent a mechanistic intermediate phenotype linking heavy drinking to the development of alcohol use disorder.

## Supplementary information

Supplementary File

## Data Availability

The data supporting the findings of this study are available from the corresponding author upon reasonable request.
