# Supplementary material for: Altered GABAB receptor sensitivity in heavy drinkers indexed using a TMS-based biomarker: a randomized controlled experimental medicine trial
Source: Transl Psychiatry. 2026 Oct 23;16:582. doi: 10.1038/s41398-026-04530-4 (PMC13691122; doi:10.1038/s41398-026-04530-4)
Supplement: Supplementary file 1 [file 41398_2026_4530_MOESM1_ESM.docx]

# Supplementary Materials

# Supplementary Methods

## Statistical Analysis

All analyses were performed in RStudio (version 4.2.3) using the *lme4, lmerTest, emmeans, afex, performance, effectsize* packages. Statistical significance was set at α = .05 (two-tailed). Effect sizes for repeated-measures ANOVA were reported as partial eta squared (η²ₚ), and estimates from linear mixed-effects models (LMMs) were reported as unstandardized regression coefficients (β) with Satterthwaite-adjusted degrees of freedom. All models included a random intercept for subjects to account for the repeated-measures, within-subject crossover design.

# Supplementary Results

|  | Light Drinkers  Female | Heavy Drinkers  Female | Light Drinkers  Male | Heavy Drinkers  Male | *p-value* |
| --- | --- | --- | --- | --- | --- |
| **n** | 7 | 8 | 8 | 8 |  |
| **Age** | 28.41 (7.98) | 22.32 (0.59) | 27.65 (10.32) | 25.75 (6.16) | 0.354 |
| **Hormonal therapy (%)** | 3 (42.9) | 5 (62.5) | 0 | 0 |  |
| **DUDIT** | 0.29 (0.76) | 0.88 (2.47) | 0.00 (0.00) | 1.25 (1.39) | 0.352 |
| **AUDIT** |  |  |  |  |  |
| Mean (SD) | 4.71 (2.06) | 13.88 (5.64) | 5.62 (2.72) | 14.12 (4.19) | **<0.001** |
| Median (IQR) | 5.00 (4.50–5.00) | 12.00 (11.75–14.75) | 6.00 (4.00–6.50) | 13.50 (10.75–18.00) |  |
| Range | 1–8 | 7–26 | 1–10 | 9–20 |  |
| **Drinks/week** |  |  |  |  |  |
| Mean (SD) | 2.75 (1.57) | 13.19 (4.38) | 5.83 (4.22) | 16.62 (8.65) | **<0.001** |
| Median (IQR) | 3.00 (1.75–3.88) | 14.00 (10.44–15.31) | 5.88 (2.19–9.28) | 15.88 (11.81–18.56) |  |
| Range | 0.25–4.75 | 7.25–19.25 | 0.50–11.00 | 5.50–34.75 |  |
| **Binge Drinking Episodes,** n (%) |  |  |  |  | **<0.001** |
| Almost every day | 0 (0) | 0 (0) | 0 (0) | 1 (12.5) |  |
| Once per week | 0 (0) | 7 (87.5) | 0 (0) | 5 (62.5) |  |
| Once per month | 1 (14.3) | 1 (12.5) | 4 (50) | 2 (25) |  |
| < once per month | 5 (71.4) | 0 (0) | 3 (37.5) | 0 (0) |  |
| Never | 1 (14.3) | 0 (0) | 1 (12.5) | 0 (0) |  |
| **Growth Hormone** | 6.21 (3.08) | 5.10 (4.33) | 0.46 (0.60) | 1.92 (3.42) | 0.004 |
| **EHI** | 7.43 (0.79) | 5.38 (3.85) | 7.00 (1.41) | 5.38 (5.58) | 0.559 |
| **Handedness** | 0 | 1 | 0 | 1 | 0.572 |
| **RMT** | 41.21 (5.16) | 46.73 (3.96) | 39.71 (5.86) | 45.35 (7.07) | 0.063 |
| **LICI100** | 35.68 (13.42) | 31.06 (17.66) | 27.41 (17.96) | 45.12 (20.39) | 0.237 |
| **SICI** | 34.32 (11.41) | 70.29 (69.56) | 33.14 (16.75) | 40.49 (30.75) | 0.265 |
| **ICF** | 141.01 (52.21) | 60.59 (30.74) | 110.27 (38.46) | 82.27 (31.51) | 0.005 |

**Table S1.** **Sex-stratified baseline demographic, clinical and neurophysiological characteristics of light (LD) and heavy drinkers (HD).** Values are mean (SD) or n (%). Weekly alcohol consumption and AUDIT scores are additionally reported as median (IQR) and full range to characterize their distributions within each sex and drinking group. P values derive from two-way ANOVA models including drinking group, sex, and their interaction (Outcome ~ Group × Sex), or Fisher's exact test where appropriate.

|  | Light Drinkers (n: 15) | Heavy Drinkers (n:16) | *p-value* |
| --- | --- | --- | --- |
| **MADRS** | 4.20 (3.36) | 6.00 (3.93) | 0.183 |
| **Behavioral Activation Scale (BAS)** |  |  |  |
| Punishment | 11.60 (2.16) | 9.38 (2.78) | **0.019** |
| Reward | 11.60 (1.76) | 10.19 (1.28) | **0.016** |
| Drive | 8.93 (1.62) | 9.12 (1.50) | 0.735 |
| Fun | 9.27 (1.22) | 8.56 (1.82) | 0.220 |
| **Barratt Impulsiveness Scale (BIS)** |  |  |  |
| Attentional Impulsiveness | 14.00 (1.77) | 15.56 (2.42) | 0.051 |
| Motor Impulsiveness | 18.93 (2.81) | 20.75 (4.02) | 0.159 |
| Non planning impulsiveness | 12.33 (2.02) | 13.75 (1.88) | 0.053 |
| **Revised NEO Personality Inventory** |  |  |  |
| Conscientiousness | 32.93 (9.88) | 37.06 (3.68) | 0.129 |
| Neuroticism | 37.00 (10.80) | 40.19 (4.75) | 0.291 |
| Extraversion | 27.47 (7.01) | 31.88 (3.01) | **0.029** |
| Agreableness | 26.13 (6.47) | 29.06 (3.38) | 0.121 |
| Openness | 24.87 (7.40) | 28.94 (2.54) | **0.047** |

## Psychometric Characteristics

**Table S2.** **Baseline psychometric characteristics of light drinkers (LD) and heavy drinkers (HD).** Values are presented as mean (SD) unless otherwise indicated. Group comparisons were performed using independent-samples t tests for continuous variables and Fisher’s exact test for categorical variables. Abbreviations: BAS = Behavioral Activation Scale; BIS = Barratt Impulsiveness Scale; MADRS = Montgomery–Åsberg Depression Rating Scale; NEO = Revised NEO Personality Inventory.

Light and heavy drinkers differed on several psychometric measures relevant to reward processing, personality, and impulsivity (see **Table S2**). Compared with HD, LD showed higher behavioral activation related to reward (LD: 11.6 ± 1.8; HD: 10.2 ± 1.3; p = .016) and punishment sensitivity (LD: 11.6 ± 2.2; HD: 9.4 ± 2.8; p = .019), as assessed by BAS subscales. In terms of personality traits, HD scored higher on extraversion (LD: 27.5 ± 7.0; HD: 31.9 ± 3.0; p = .030) and openness (LD: 24.9 ± 7.4; HD: 28.9 ± 2.5; p = .047) on the Revised NEO Personality Inventory. Additionally, HD showed trend-level increases in attentional impulsivity (LD: 14.0 ± 1.8; HD: 15.6 ± 2.4; p = .051) and non-planning impulsivity (LD: 12.3 ± 2.0; HD: 13.8 ± 1.9; p = .053), as measured by the Barratt Impulsiveness Scale. These differences align with established behavioral phenotypes associated with heavier alcohol consumption and provide further context for the observed neurophysiological group differences.

Cortical Excitability Measures

|  | Light Drinkers | | | Heavy Drinkers | | |
| --- | --- | --- | --- | --- | --- | --- |
|  | **PBO** | **BAC30** | **BAC60** | **PBO** | **BAC30** | **BAC60** |
| **RMT** | | | | | | |
| Baseline | 40.82 (5.17) | 45.67 (5.63) | 40.26 (5.50) | 41.38 (5.53) | 46.97 (5.92) | 45.99 (5.48) |
| 1-hour | 40.72 (5.33) | 40.92 (5.75) | 40.41 (5.13) | 45.58 (6.46) | 46.97 (5.05) | 46.68 (6.11) |
| **MEP amplitude for SP 130%** | | | | | | |
| Baseline | 361.22 (363.25) | 267.23 (168.12) | 194.68 (103.17) | 180.91 (102.71) | 168.84 (92.89) | 164.06 (81.59) |
| 1-hour | 296.89 (227.73) | 285.99 (174.79) | 217.45 (91.81) | 198.45 (99.19) | 184.92 (95.27) | 131.87 (52.39) |
| **LICI** | | | | | | |
| Baseline | 24.39 (12.61) | 28.28 (16.89) | 26.58 (12.86) | 28.71 (16.98) | 31.96 (15.48) | 34.72 (22.62) |
| 1-hour | 25.86 (14.75) | 18.31 (10.26) | 16.92 (7.53) | 25.24 (14.45) | 25.22 (14.50) | 35.94 (21.33) |
| **SICI** | | | | | | |
| Baseline | 48.02 (30.10) | 34.74 (18.25) | 37.72 (24.44) | 38.44 (25.02) | 44.57 (25.21) | 58.61 (54.25) |
| 1-hour | 45.84 (27.03) | 41.21 (28.32) | 33.35 (22.38) | 37.80 (23.05) | 61.56 (41.06) | 51.57 (33.71) |
| **ICF** | | | | | | |
| Baseline | 103.28 (42.41) | 113.93 (29.67) | 124.40 (67.69) | 84.35 (40.65) | 96.12 (66.83) | 101.86 (56.59) |
| 1-hour | 145.25 (54.58) | 95.79 (27.48) | 85.83 (38.28) | 93.61 (51.54) | 93.91 (52.73) | 112.39 (41.37) |

**Table S3. Cortical excitability measures in light drinkers (LD) and heavy drinkers (HD) across placebo, baclofen 30 mg, and baclofen 60 mg conditions.** Values represent mean (SD). Measures of corticospinal excitability (resting motor threshold [RMT] and single-pulse MEP amplitude), GABAB-mediated inhibition (LICI), GABAA-mediated inhibition (SICI), and intracortical facilitation (ICF) were collected at baseline and 1 hour after drug administration, corresponding to peak baclofen exposure. Across groups, placebo produced minimal changes in cortical excitability. Baclofen produced dose-dependent reductions in ICF and LICI in LD, consistent with enhanced GABAB-mediated inhibition, whereas HD showed attenuated or inconsistent modulation across doses. SICI exhibited no systematic drug-related changes in either group. Abbreviations: RMT = resting motor threshold; MEP = motor-evoked potential; LICI = long-interval intracortical inhibition; SICI = short-interval intracortical inhibition; ICF = intracortical facilitation; PBO = placebo; BAC30 = baclofen 30 mg; BAC60 = baclofen 60 mg.

## Baclofen and Growth Hormone Plasma Levels

|  | PBO | BAC30 | BAC60 | PBO | BAC30 | BAC60 |
| --- | --- | --- | --- | --- | --- | --- |
| **Growth Hormone** |  |  |  |  |  |  |
| Baseline | 3.81 (3.56) | 2.30 (3.51) | 1.86 (2.28) | 3.99 (4.79) | 3.93 (4.12) | 3.32 (4.78) |
| 1-hour after drug | 0.90 (0.85) | 1.56 (2.20) | 4.57 (8.68) | 0.60 (0.57) | 1.03 (0.72) | 1.37 (1.48) |
| 2-hour after drug | 0.48 (0.38) | 7.27 (7.06) | 14.12 (12.35) | 0.74 (1.30) | 5.84 (5.41) | 7.79 (5.74) |
| **Baclofen** |  |  |  |  |  |  |
| 1-hour after drug | 0.00 (0.00) | 0.24 (0.11) | 0.48 (0.21) | 0.00 (0.00) | 0.29 (0.12) | 0.46 (0.27) |
| 2-hour after drug | 0.00 (0.00) | 0.30 (0.06) | 0.56 (0.20) | 0.00 (0.00) | 0.37 (0.08) | 0.58 (0.23) |

**Table S4. Growth hormone (GH) plasma concentrations and baclofen blood levels in light drinkers (LD) and heavy drinkers (HD) across treatment conditions.** Values are presented as mean (SD). GH was measured at baseline, 1 hour, and 2 hours after administration of placebo, baclofen 30 mg, or baclofen 60 mg. Baclofen plasma concentrations were assessed at 1 and 2 hours post-administration. GH levels showed minimal change following placebo in both groups. In contrast, baclofen produced dose-dependent increases in GH, particularly at 60 mg, with larger elevations observed in HD relative to LD at 2 hours post-dose. Blood baclofen concentrations increased proportionally with dose and time, and were comparable across drinking groups. Abbreviations: GH = growth hormone; PBO = placebo; BAC30 = baclofen 30 mg; BAC60 = baclofen 60 mg.

| **Effect** | ***df (num, den)*** | ***F*** | ***p value*** | ***η²ₚ*** |
| --- | --- | --- | --- | --- |
| Group | 1,21 | 0.03 | 0.858 | 0.002 |
| Treatment | 2,42 | 127.42 | 0.000 | 0.860 |
| Timepoint | 2,42 | 151.16 | 0.000 | 0.880 |
| Group × Treatment | 2,42 | 0.80 | 0.402 | 0.040 |
| Group × Timepoint | 2,42 | 0.77 | 0.450 | 0.040 |
| Treatment × Timepoint | 4,84 | 73.09 | 0.000 | 0.780 |
| Group × Treatment × Timepoint | 4,84 | 0.83 | 0.464 | 0.040 |

**Table S5.** **Baclofen plasma concentration.** Values are from a Type III repeated-measures ANOVA with Greenhouse–Geisser correction applied where appropriate. Treatment (placebo, baclofen 30 mg, baclofen 60 mg) and timepoint (baseline, 1 hour post-drug and 2 hours post-drug) were within-subject factors; group (heavy vs light drinkers) was between-subject factor. Effect sizes are reported as partial eta squared (η²ₚ).

## Effects of Baclofen on Growth Hormone (GH)

| Effect | *df (num, den)* | *F* | *p value* | η²ₚ |
| --- | --- | --- | --- | --- |
| Group | 1, 25 | 2.7 | 0.113 | 0.10 |
| Treatment | 2, 50 | 17.54 | <0.01 | 0.41 |
| Group × Treatment | 2, 50 | 4.07 | 0.042 | 0.14 |
| Timepoint | 2, 50 | 16.57 | <0.01 | 0.40 |
| Group × Timepoint | 2, 50 | 4.30 | 0.0367 | 0.15 |
| Treatment × Timepoint | 4, 100 | 4.30 | <0.01 | 0.37 |
| Group × Treatment × Timepoint | 4, 100 | 1.77 | 0.142 | 0.07 |

**Table S6.** **Baclofen effects on GH.** Values are from a Type III repeated-measures ANOVA with Greenhouse–Geisser correction applied where appropriate. Treatment (placebo, baclofen 30 mg, baclofen 60 mg) and timepoint (baseline, 1 h and 2 h post-drug) were within-subject factors; group (heavy vs light drinkers) was a between-subject factor. Effect sizes are reported as partial eta squared (η²ₚ).

### Post-hoc analyses

To characterize the temporal and dose-dependent effects of baclofen on GH secretion in heavy drinkers (HD) and light drinkers (LD), post-hoc pairwise comparisons were conducted following the repeated-measures ANOVA. All contrasts were Benjamini–Hochberg (BH)–adjusted for multiple comparisons. Collectively, post-hoc analyses confirmed a clear dose- and time-dependent GH response to baclofen, with attenuated sensitivity in heavy drinkers. The divergence between groups was most pronounced at the highest baclofen dose and latest time point, consistent with altered presynaptic GABA**_B_**Rs–mediated endocrine responsiveness in individuals with heavy alcohol consumption. Under placebo, GH levels declined over time in heavy drinkers (baseline vs 1 h: *p* = 0.007; baseline vs 2 h: *p* = 0.011), whereas no significant changes were observed in light drinkers (all *p* > 0.13). Following baclofen 30 mg, heavy drinkers showed a reduction in GH from baseline to 1 h (*p* = 0.007), followed by a relative increase from 1 h to 2 h (*p* = 0.026), resulting in no significant difference between baseline and 2 h (*p* = 0.44). In light drinkers, GH increased significantly from baseline to 2 h (*p* = 0.031) and from 1 h to 2 h (*p* = 0.008), while the baseline-to-1 h comparison was not significant (*p* = 0.51). After baclofen 60 mg, a pronounced delayed increase in GH was observed. In light drinkers, GH rose significantly from baseline to 2 h (*p* < 0.001) and from 1 h to 2 h (*p* < 0.001), whereas the baseline-to-1 h contrast was not significant (*p* = 0.15). In heavy drinkers, GH increased significantly from 1 h to 2 h (*p* = 0.007), although neither baseline-to-1 h nor baseline-to-2 h comparisons reached statistical significance (both *p* > 0.17). Between-group contrasts were not significant at 30 mg. At 60 mg, light drinkers exhibited numerically greater late GH increases (*p* = 0.069), consistent with the significant group × treatment interaction observed in the omnibus model.

## Effects of Baclofen on Long-Interval Intracortical Inhibition

### Pre-registered repeated measures ANOVA

| **Effect** | ***df (num, den)*** | ***F*** | ***p value*** | ***η²ₚ*** |
| --- | --- | --- | --- | --- |
| Group | 1,29 | 2.37 | 0.134 | 0.08 |
| Treatment | 2,58 | 0.87 | 0.423 | 0.03 |
| Group × Treatment | 2,58 | 3.68 | 0.039 | 0.11 |
| Timepoint | 1,29 | 11.65 | 0.002 | 0.29 |
| Group × Timepoint | 1,29 | 1.33 | 0.258 | 0.04 |
| Treatment × Timepoint | 2,58 | 2.92 | 0.063 | 0.09 |
| Group × Treatment × Timepoint | 2,58 | 3.36 | 0.042 | 0.10 |

**Table S7. Baclofen effects on Long-Intracortical Inhibition (LICI) by group.** Values are from a Type III repeated-measures ANOVA with Greenhouse–Geisser correction applied where appropriate. Treatment (placebo, baclofen 30 mg, baclofen 60 mg) and timepoint (baseline, 1 h post-drug) were within-subject factors; group (heavy vs light drinkers) was a between-subject factor. Effect sizes are reported as partial eta squared (η²ₚ).

### Post-hoc analyses

BH–adjusted pairwise comparisons were performed to further characterize the significant treatment × group × time interaction observed in the repeated-measures ANOVA. No significant between-group differences were detected at baseline across treatment sessions (all adjusted *p* > 0.10), confirming comparable pre-drug inhibitory tone. Under placebo, heavy drinkers showed a modest reduction in LICI from baseline to 1 h (adjusted *p* = 0.015) and from baseline to 2 h (adjusted *p* = 0.011), whereas light drinkers did not exhibit significant time-dependent changes (all adjusted *p* > 0.10). Following baclofen 30 mg, both groups demonstrated significant increases in LICI at 1 h relative to baseline (light: adjusted *p* = 0.005; heavy: adjusted *p* = 0.043).

After baclofen 60 mg, light drinkers exhibited a significant increase in LICI at 1 h compared with baseline (adjusted *p* = 0.014). In contrast, heavy drinkers showed no significant increase in inhibition at either post-drug timepoint (baseline vs 1 h: adjusted *p* = 0.73; baseline vs 2 h: adjusted *p* > 0.10). Consequently, a significant between-group difference emerged at 1 h following 60 mg (adjusted *p* = 0.003), reflecting preserved dose-dependent enhancement in light drinkers and a flattened response in heavy drinkers. Together, these BH-adjusted analyses confirm that group differences were not present at baseline but emerged selectively under high-dose baclofen, supporting a dose-dependent divergence in GABABRs-mediated cortical inhibition.

### Sensitivity analyses: Sex, Visit Order and Baclofen Concentrations Effects on Main Model

| **Effect** | ***df (num, den)*** | ***F*** | ***p value*** | ***η²ₚ*** |
| --- | --- | --- | --- | --- |
| Group | 1,28 | 2.36 | 0.135 | 0.08 |
| Sex | 1,28 | 0.28 | 0.601 | 0.01 |
| Treatment | 2,56 | 0.834 | 0.423 | 0.03 |
| Timepoint | 1,28 | 11.34 | 0.002 | 0.29 |
| Group × Treatment | 2,56 | 3.56 | 0.043 | 0.11 |
| Sex × Treatment | 2,56 | 0.05 | 0.934 | 0.00 |
| Group × Timepoint | 1,28 | 1.25 | 0.273 | 0.04 |
| Sex × Timepoint | 1,28 | 0.77 | 0.390 | 0.03 |
| Treatment × Timepoint | 2,56 | 2.80 | 0.071 | 0.09 |
| Group × Treatment × Timepoint | 2,56 | 3.21 | 0.049 | 0.1 |
| Sex × Treatment × Timepoint | 2,56 | 0.23 | 0.792 | 0.01 |

**Table S8.** **Baclofen effects on long-intracortical inhibition (LICI) by group and sex.** Values are from a Type III repeated-measures ANOVA with Greenhouse–Geisser correction applied where appropriate. Treatment (placebo, baclofen 30 mg, baclofen 60 mg) and timepoint (baseline, 1 h post-drug) were within-subject factors; group (heavy vs light drinkers) and sex (male, female) were between-subject factors. Effect sizes are reported as partial eta squared (η²ₚ).

| **Effect** | ***df (num, den)*** | ***F*** | ***p value*** | ***η²ₚ*** |
| --- | --- | --- | --- | --- |
| Group | 1,24 | 2.44 | 0.131 | 0.09 |
| Sequence | 5,24 | 0.94 | 0.472 | 0.16 |
| Treatment | 1.54, 37.03 | 1.43 | 0.250 | 0.06 |
| Group × Treatment | 1.54, 37.03 | 4.19 | 0.032 | 0.33 |
| Sequence × Treatment | 7.71, 37.03 | 1.81 | 0.108 | 0.15 |
| Timepoint | 1,24 | 11.82 | 0.002 | 0.27 |
| Group × Timepoint | 1,24 | 1.30 | 0.265 | 0.05 |
| Sequence × Timepoint | 5,24 | 0.45 | 0.811 | 0.09 |
| Treatment × Timepoint | 1.97, 47.28 | 2.22 | 0.120 | 0.08 |
| Group × Treatment × Timepoint | 1.97, 28 | 3.04 | 0.058 | 0.11 |
| Sequence × Treatment × Timepoint | 9.85, 47.28 | 0.97 | 0.478 | 0.17 |

**Table S9.** **Baclofen effects on long-intracortical inhibition (LICI) by group and visit order.** Values are from a Type III repeated-measures ANOVA with Greenhouse–Geisser correction applied where appropriate. Treatment (placebo, baclofen 30 mg, baclofen 60 mg) and timepoint (baseline, 1 h post-drug) were within-subject factors; group (heavy vs light drinkers) and treatment sequence (Williams square order) were between-subject factors. Effect sizes are reported as partial eta squared (η²ₚ).

| Effect | Df (num, den) | F | p | partial η² |
| --- | --- | --- | --- | --- |
| Treatment | 2, 131 | 0.762 | 0.469 | 0.01 |
| Group | 1, 28 | 3.647 | 0.067 | 0.12 |
| Timepoint | 1, 134 | 3.016 | 0.085 | 0.02 |
| Plasma baclofen concentration | 1, 137 | 0.007 | 0.934 | <0.001 |
| Treatment × Group | 2, 129 | 3.841 | 0.024 | 0.06 |
| Treatment × Timepoint | 2, 130 | 1.505 | 0.226 | 0.02 |
| Group × Timepoint | 1, 127 | 0.629 | 0.429 | <0.01 |
| Treatment × Group × Timepoint | 2, 127 | 1.979 | 0.142 | 0.03 |

**Table S10.** **Sensitivity analysis of LICI responses adjusted for plasma baclofen concentration.** Type III tests from a linear mixed-effects model examining long-interval intracortical inhibition (LICI) as a function of treatment, drinking group, timepoint, and their interactions, with individual plasma baclofen concentration included as a covariate and participant as a random intercept. Degrees of freedom were estimated using the Kenward–Roger method. Effect sizes are reported as partial eta squared (η²p). Plasma baclofen concentration was not significantly associated with LICI, whereas the treatment × group interaction remained significant after adjustment for individual differences in systemic baclofen exposure.

### Baseline-adjusted Delta Analysis

| Effect | *df (num, den)* | | *F* | | *p value* |
| --- | --- | --- | --- | --- | --- |
| **Model: baseline LICI-adjusted** |  |  |  |  |  |
| Treatment | 2,57 | | 3.94 | | 0.025 |
| Group | 1,28 | | 1.99 | | 0.169 |
| Baseline LICI | 1,28 | | 15.44 | | 0.000 |
| Group × Treatment | 2,57 | | 6.17 | | 0.004 |
| **Model: baseline RMT-adjusted** |  |  | |  |  |
| Treatment | 2, 58 | | 2.84 | | 0.066 |
| Group | 1, 28 | | 2.83 | | 0.103 |
| Baseline LICI | 1, 49 | | 32.72 | | 0.000 |
| Baseline RMT | 1, 32 | | 0.91 | | 0.348 |
| Group × Treatment | 2, 58 | | 5.27 | | 0.008 |

**Table S11.** **Sensitivity analysis of baclofen-induced changes in LICI adjusted for baseline LICI and RMT.** Values are from ANCOVA, with Kenward-Roger method for degrees of freedom adjustment applied whether appropriate. Treatment (placebo, baclofen 30 mg, baclofen 60 mg) was within-subject factors; group (heavy vs light drinkers) was between-subject factors. Baseline LICI and RMT account for measures before drug administration. Delta values account for LICI differences, among conditions, per both groups.

| Model / Effect | *df (num, den)* | *F* | *p value* |
| --- | --- | --- | --- |
| **Model 1: AUDIT score** |  |  |  |
| Drug | 2,58.09 | 2.41 | 0.099 |
| AUDIT | 1,28.46 | 2.25 | 0.144 |
| Baseline LICI | 1,48.72 | 26.90 | <0.001 |
| Drug × AUDIT | 2,57.64 | 1.96 | 0.151 |
| **Model 2: Weekly alcohol consumption** |  |  |  |
| Drug | 2,58.09 | 2.35 | 0.104 |
| Drinks/week | 1,28.55 | 0.93 | 0.344 |
| Baseline LICI | 1,49.11 | 24.96 | <0.001 |
| Drug × Drinks/week | 2, 57.61 | 1.10 | 0.338 |

**Table S12. Exploratory dimensional analyses of baclofen-induced changes LICI.** Type III tests from linear mixed-effects models examining absolute change in LICI (ΔLICI = 1 h − baseline) as a function of drug and either AUDIT score (Model 1) or average weekly alcohol consumption (Model 2), entered as continuous variables. Both models included session-specific baseline LICI as a covariate and participant as a random intercept. Continuous alcohol-use variables were mean-centered. Neither AUDIT score nor weekly alcohol consumption significantly moderated the effect of baclofen on ΔLICI.

### Post-hoc analyses

To further characterize drug effects independent of baseline variability, we analyzed change-from-baseline LICI (ΔLICI) using pairwise contrasts within each drinking group. In light drinkers, both active doses of baclofen significantly increased inhibition relative to placebo. Specifically, ΔLICI was greater following baclofen 30 mg compared with placebo (estimate = 9.92, t(58) = 2.60, p = 0.018) and following baclofen 60 mg compared with placebo (estimate = 10.27, t(57.7) = 2.70, p = 0.018). No significant difference was observed between baclofen 30 mg and 60 mg (estimate = 0.35, t(57.6) = 0.09, p = 0.93), indicating a comparable magnitude of drug-induced inhibition across doses in this group. In heavy drinkers, baclofen 30 mg did not differ from placebo (estimate = 1.99, t(57.8) = 0.54, p = 0.59). The comparison between placebo and baclofen 60 mg showed a trend-level effect (estimate = −7.06, t(58.6) = −1.91, p = 0.092), suggesting a reduction in inhibition at the higher dose. Moreover, the contrast between baclofen 30 mg and 60 mg approached statistical significance (estimate = −9.05, t(57.8) = −2.46, p = 0.051), consistent with a dose-dependent divergence in response within heavy drinkers. Together, the Δ analysis confirms a robust baclofen-induced increase in cortical inhibition in light drinkers at both doses, whereas heavy drinkers failed to show significant enhancement and exhibited a trend toward reduced inhibition at the higher dose.

### Relationships between drug induced changes in LICI and GH

Changes in LICI were not significantly correlated with changes in growth hormone in either group under placebo, 30-mg baclofen, or 60-mg baclofen conditions (all $\mid r\mid\leq.20$, all $p\geq.47$). Notably, no association was observed at 60 mg baclofen in either light drinkers ($r=.08, p=.798$) or heavy drinkers ($r=-.02, p=.947$), supporting a dissociation between cortical and neuroendocrine GABA_B_-mediated responses.

### Secondary cortical excitability measures

| Effect | *df (num, den)* | *F* | *p value* | η²ₚ |
| --- | --- | --- | --- | --- |
| **SICI** |  |  |  |  |
| Group | 1, 20 | 0.24 | 0.630 | 0.01 |
| Treatment | 2, 40 | 0.36 | 0.700 | 0.02 |
| Group × Treatment | 2, 40 | 2.17 | 0.130 | 0.10 |
| Timepoint | 1, 20 | 4.04 | 0.058 | 0.17 |
| Group × Timepoint | 1, 20 | 4.28 | 0.051 | 0.18 |
| Treatment × Timepoint | 2, 40 | 0.34 | 0.650 | 0.02 |
| Group × Treatment × Timepoint | 2, 40 | 0.037 | 0.964 | <0.01 |
| **ICF** |  |  |  |  |
| Group | 1, 23 | 2.21 | 0.151 | 0.09 |
| Treatment | 2, 46 | 0.48 | 0.621 | 0.02 |
| Group × Treatment | 2, 46 | 1.24 | 0.299 | 0.05 |
| Timepoint | 1, 23 | 0.002 | 0.969 | <0.01 |
| Group × Timepoint | 1, 23 | 0.53 | 0.474 | 0.02 |
| Treatment × Timepoint | 2, 46 | 4.52 | 0.016 | 0.16 |
| Group × Treatment × Timepoint | 2, 46 | 2.05 | 0.14 | 0.08 |
| **RMT** |  |  |  |  |
| Group | 1, 29 | 8.31 | 0.007 | 0.22 |
| Treatment | 2, 46 | 2.37 | 0.100 | 0.08 |
| Group × Treatment | 2, 46 | 1.04 | 0.361 | 0.03 |
| Timepoint | 1, 23 | 0.03 | 0.873 | <0.01 |
| Group × Timepoint | 1, 23 | 0.75 | 0.392 | 0.03 |
| Treatment × Timepoint | 2, 46 | 1.20 | 0.310 | 0.04 |
| Group × Treatment × Timepoint | 2, 46 | 0.20 | 0.813 | <0.01 |

**Table S13.** **Baclofen effects on short-intracortical inhibition (SICI).** Values are from a Type III repeated-measures ANOVA with Greenhouse–Geisser correction applied where appropriate. Treatment (placebo, baclofen 30 mg, baclofen 60 mg) and timepoint (baseline, 1 h post-drug) were within-subject factors; group (heavy vs light drinkers) was a between-subject factors. Effect sizes are reported as partial eta squared (η²ₚ).
